# Descriptors of Sepsis Using the Sepsis-3 Criteria: A Cohort Study in Critical Care Units Within the U.K. National Institute for Health Research Critical Care Health Informatics Collaborative*

**DOI:** 10.1097/CCM.0000000000005169

**Published:** 2021-07-01

**Authors:** Anoop D. Shah, Niall S. MacCallum, Steve Harris, David A. Brealey, Edward Palmer, James Hetherington, Sinan Shi, David Perez-Suarez, Ari Ercole, Peter J. Watkinson, Andrew Jones, Simon Ashworth, Richard Beale, Stephen J. Brett, Mervyn Singer

**Affiliations:** 1 University College London Hospitals NHS Foundation Trust, London, United Kingdom.; 2 University College London Hospitals Biomedical Research Centre, London, United Kingdom.; 3 Institute of Health Informatics, University College London, London, United Kingdom.; 4 Bloomsbury Institute of Intensive Care Medicine, University College London, London, United Kingdom.; 5 Research Software Development Group, Research IT Services, University College London, London, United Kingdom.; 6 Department of Statistics, University of Oxford, Oxford, United Kingdom.; 7 Division of Anaesthesia, University of Cambridge, Cambridge, United Kingdom.; 8 Nuffield Department of Clinical Neurosciences, University of Oxford, John Radcliffe Hospital, Oxford, United Kingdom.; 9 Department of Intensive Care, Guy’s and St. Thomas’ NHS Foundation Trust, London, United Kingdom.; 10 Centre for Perioperative Medicine and Critical Care Research, Imperial College Healthcare NHS Trust, London, United Kingdom.; 11 Department of Surgery and Cancer, Imperial College London, London, United Kingdom.; 12 Centre for Perioperative Medicine and Critical Care Research, Imperial College Healthcare NHS Trust, London, United Kingdom.

**Keywords:** antibiotics, electronic health records, sepsis-3, septic shock

## Abstract

To describe the epidemiology of sepsis in critical care by applying the Sepsis-3 criteria to electronic health records.

**DESIGN:**

Retrospective cohort study using electronic health records.

**SETTING:**

Ten ICUs from four U.K. National Health Service hospital trusts contributing to the National Institute for Health Research Critical Care Health Informatics Collaborative.

**PATIENTS:**

A total of 28,456 critical care admissions (14,332 emergency medical, 4,585 emergency surgical, and 9,539 elective surgical).

**MEASUREMENTS AND MAIN RESULTS:**

Twenty-nine thousand three hundred forty-three episodes of clinical deterioration were identified with a rise in Sequential Organ Failure Assessment score of at least 2 points, of which 14,869 (50.7%) were associated with antibiotic escalation and thereby met the Sepsis-3 criteria for sepsis. A total of 4,100 episodes of sepsis (27.6%) were associated with vasopressor use and lactate greater than 2.0 mmol/L, and therefore met the Sepsis-3 criteria for septic shock. ICU mortality by source of sepsis was highest for ICU-acquired sepsis (23.7%; 95% CI, 21.9–25.6%), followed by hospital-acquired sepsis (18.6%; 95% CI, 17.5–19.9%), and community-acquired sepsis (12.9%; 95% CI, 12.1–13.6%) (*p* for comparison less than 0.0001).

**CONCLUSIONS:**

We successfully operationalized the Sepsis-3 criteria to an electronic health record dataset to describe the characteristics of critical care patients with sepsis. This may facilitate sepsis research using electronic health record data at scale without relying on human coding.

## Abstract 2

Supplemental Digital Content is available in the text.

Sepsis is a leading cause of mortality and critical illness worldwide (1) and a common reason for admission to ICUs, but it is often hard to identify, with no reliable diagnostic test (2). Sepsis is defined as a dysregulated and deleterious host response to infection leading to organ dysfunction (3), though this represents an umbrella syndrome covering a host of biological and clinical phenotypes.

The original 1992 criteria for sepsis (4, 5) were based on the presence of two or more Systemic Inflammatory Response Syndrome criteria related to suspected or proven infection. However, ill-defined criteria for organ dysfunction (“severe sepsis”) and septic shock led to a reported incidence and mortality rate that could vary three- to 10-fold (6). The 2016 “Sepsis-3” Task Force (3) aimed to improve the consistency of reporting by offering specific clinical criteria that characterized organ dysfunction and shock with a clearer association with mortality. Sepsis-3 uses a change in the Sequential Organ Failure Assessment (SOFA) score of 2 or more points associated with the acute infectious episode as the clinical criterion for new organ dysfunction (7, 8).

It has been difficult to describe the epidemiology of sepsis using routine data because clinical coding data do not capture all cases (9, 10) and are affected by coding practices that have changed over time (11, 12). Objective definitions of sepsis based on clinical parameters in electronic health records (EHRs) have been found to provide more stable disease estimates over time than coding data (13, 14). Such studies rely on detailed hospital health records being available for research at scale, which has not previously been the case in the United Kingdom.

In this study, we sought to describe the epidemiology of sepsis and patterns of antibiotic use in ICUs by operationalizing the Sepsis-3 definitions within EHRs. We used data from ICUs within four large National Health Service (NHS) Hospital Trusts with Biomedical Research Centers, which contribute to the National Institute of Health Research (NIHR) Critical Care theme of the Health Informatics Collaborative (CC-HIC).

## MATERIALS AND METHODS

### Study Population

CC-HIC was established in 2013 to facilitate use of routinely collected ICU data for research. The CC-HIC research platform has been described previously (15, 16); it harmonizes EHR data from multiple hospitals, including demographics, physiology, organ support, medication, and outcomes (17). It also includes summary data about ICU admissions (such as diagnoses) submitted to the Intensive Care National Audit and Research Centre (ICNARC) Case Mix Program (18). Data are stored securely within the ISO 27001 certified Data Safe Haven at University College London. CC-HIC has been approved by the Caldicott guardians of the contributing NHS Trusts, the National Research Ethics Service (14/LO/1031), and the Confidentiality Advisory Group of the Health Research Authority.

All adult ICU admissions (18 yr and older) in four participating NHS Trusts from February 2014 to July 2018 were eligible for inclusion. We excluded patients who had opted out of use of their data for research, those with inadequate data quality, and those who lacked physiologic and laboratory data to calculate three or more SOFA parameters. We followed up patients using data recorded in their EHR during the period of their ICU admission.

### Identification of Infection

For the purpose of applying the Sepsis-3 criteria, we defined infection as a new course of antibiotics or an escalation in antibiotic therapy, with at least one antibiotic given intravenously. Antibiotics were ranked according to the classification of Braykov et al (19), which represents their activity against drug-resistant organisms. “Antibiotic escalation” was defined as an increase in the maximum rank of out of all current antibiotics from one 24-hour period to the next or an increase in the number of antibiotics prescribed with the same maximum rank.

We considered the source of infection to be community-acquired if the patient had been admitted to hospital less than 48 hours previously, hospital-acquired if an inpatient for at least 48 hours but in ICU less than 48 hours, and ICU-acquired if they developed the infection more than 48 hours after admission to ICU.

For comparison with coded data, we report whether any of the admission diagnoses were classified as infection (x.x.x.27.x), septicemia (x.9.1.27.4), or septic shock (x.2.12.35.2) according to the ICNARC coding system (18) (where ”x” represents any number). These classifications were carried out by audit clerks as part of data submission for the national intensive care audit. However, our dataset did not include the *International Classification of Diseases*, 10th Edition (ICD-10 [20])-coded diagnosis data used for hospital reimbursement.

### Identification of Organ Dysfunction

The Sepsis-3 definitions use a change in SOFA score (7) of 2 or more points associated with the acute infectious episode as the clinical criterion for new organ dysfunction. Each organ system (cardiovascular, respiratory, renal, coagulation, liver, and CNS) is assigned a score between 0 and 4 depending on the degree of physiologic abnormality or clinical intervention. We assumed that the SOFA score was zero preadmission to ICU and zero for a particular organ system if data for that organ system were missing. Although some patients with chronic conditions would score preillness SOFA points, the fact they are being admitted to intensive care implies a significant deterioration of organ function. The overall SOFA score for each 24-hour period was the sum of the maximum value during that period for each SOFA component. More details on the calculation of SOFA scores are in **Supplemental Digital Content 1** (http://links.lww.com/CCM/G524).

### Identification of Sepsis and Septic Shock

Sepsis was operationalized by an increase in SOFA score of at least 2 points with a new antibiotic prescription or antibiotic escalation and with at least one antibiotic given intravenously. We assumed that elective surgical patients on antibiotics when admitted to ICU were receiving antibiotics as prophylaxis and were therefore not classified as sepsis regardless of their SOFA score (which was likely due to the surgery).

Septic shock was defined as sepsis with the administration of vasopressor medication and a blood lactate greater than 2 mmol/L (6).

### Statistical Analysis

We calculated descriptive statistics for characteristics and outcomes of patients by infection status at admission to critical care and for infections developing subsequently. *T* tests were used to compare means of normally distributed variables and Wilcoxon tests to compare nonnormally distributed variables. The incidence of sepsis was calculated using a Poisson model. Cumulative incidence curves were plotted by infection status at admission, treating discharge and death as competing risks. We estimated age- and sex-adjusted hazard ratios by admission sepsis status and report both cause-specific hazards from a Cox model and subdistribution hazards from a competing risks regression (Fine and Gray model). We calculated the relative use of different antibiotics by calendar time and over the course of an ICU admission. We carried out sensitivity analyses ignoring periods of norepinephrine use less than 6 hours (as these patients may not actually have required vasopressors) and ignoring Glasgow Coma Score (GCS) measurements made within 24 hours of administration of sedative medication. Data were analyzed using R 3.6.1 (R Foundation for Statistical Computing, Vienna, Austria) (21). Additional details and Strengthening the Reporting of Observational Studies in Epidemiology statement are provided in Supplemental Digital Content 1 (http://links.lww.com/CCM/G524).

## RESULTS

### Characteristics of Study Population

Adequate quality data were available from 10 ICUs in four hospitals from February 2014 to April 2018, comprising 28,786 critical care admissions (24,719 patients) (**sTable 1**, Supplemental Digital Content 2, http://links.lww.com/CCM/G525). Of the eligible admissions (14,592 emergency medical, 4,616 emergency surgical, 9,578 elective surgical), 330 had fewer than three SOFA dimensions recorded in the first 24 hours and were excluded (**sFig. 1**, Supplemental Digital Content 3, http://links.lww.com/CCM/G526).

Median age at admission was 63.1 years (interquartile range [IQR], 49.3–73.8), and 42.1% were women. Over half of the ICU admissions (16,040, 56.4%) were within 48 hours of admission to hospital (**Table 1**).

**TABLE 1.** Characteristics of ICU Admissions by Infection Status

Infection Status at Admission	Septic Shock	Sepsis Without Shock	Antibiotics Without Sepsis	Not on Antibiotics	Overall
Number of admissions	3,353	7,965	5,558	11,580	28,456
Women, *n* (%)	1,290 (38.5)	3,414 (42.9)	2,365 (42.6)	4,897 (42.3)	1,1966 (42.1)
Age, median (IQR)	63 (48.8–74.1)	61.4 (47.2–74.0)	63.3 (51.4–72.3)	63.9 (50–74.3)	63.1 (49.3–73.8)
Admission category, *n* (%)					
Elective surgical	0	0	4,498 (80.9)	5,041 (43.5)	9,539 (33.5)
Emergency surgical	782 (23.3)	1,912 (24.0)	352 (6.3)	1,539 (13.3)	4,585 (16.1)
Emergency medical	2,571 (76.7)	6,053 (76.0)	708 (12.7)	5,000 (43.2)	14,332 (50.4)
In hospital less than 48 hr prior	2,038 (60.8)	4,158 (52.2)	3,064 (55.1)	6,780 (58.5)	16,040 (56.4)
ICNARC code for infection at admission	1,289 (38.4)	3,254 (40.9)	598 (10.8)	675 (5.8)	5,816 (20.4)
Organ system affected at admission (ICNARC admission diagnosis), *n* (%)					
Cardiovascular	755 (22.8)	900 (11.4)	655 (11.9)	4,170 (36.2)	6,480 (22.9)
Respiratory	887 (26.8)	2,865 (36.2)	844 (15.3)	1,436 (12.5)	6,032 (21.3)
Hematologic	80 (2.4)	233 (2.9)	30 (0.5)	140 (1.2)	483 (1.7)
Genitourinary	268 (8.1)	801 (10.1)	1,241 (22.5)	1,332 (11.6)	3,642 (12.9)
Neurologic	260 (7.9)	688 (8.7)	298 (5.4)	1,088 (9.4)	2,334 (8.3)
Gastrointestinal	545 (16.5)	1,308 (16.5)	1,726 (31.3)	1,360 (11.8)	4,939 (17.5)
Metabolic or poisoning	102 (3.0)	398 (5.0)	156 (2.8)	1,160 (10.0)	1,816 (6.4)
Trauma	339 (10.2)	332 (4.2)	57 (1.0)	556 (4.8)	1,284 (4.5)
Other	74 (2.2)	394 (4.9)	506 (9.1)	277 (2.4)	1,251 (4.4)
First 24-hr physiology					
Maximum heart rate, median (IQR)	110 (95–126)	103 (90–118)	95 (83–107)	93 (82–105)	97 (85–112)
Minimum mean arterial pressure, mm Hg, median (IQR)	58.5 (53–63)	64 (58–71)	64 (58–71)	65 (59–73)	63 (57–71)
Maximum Fio_2_, median (IQR)	0.60 (0.41–0.95)	0.40 (0.30–0.60)	0.35 (0.28–0.45)	0.40 (0.28–0.55)	0.40 (0.28–0.60)
Minimum Spo_2_, median (IQR)	92 (88–95)	92 (89–95)	94 (92–95)	94 (92–96)	93 (91–95)
Minimum Pao_2_, mm Hg, median (IQR)	6.8 (5.0–9.6)	8.2 (5.7–9.9)	9.4 (5.7–11.2)	8.8 (5.2–10.8)	8.5 (5.4–10.5)
Minimum Pao_2_:Fio_2_ ratio, median (IQR)	16 (10–25)	22 (14–33.7)	32 (19.6–44)	26 (15–41)	24 (15–38)
Minimum Glasgow Coma Score, median (IQR)	6 (3–13)	14 (8–15)	14 (10–15)	14 (6–15)	14 (6–15)
Maximum creatinine in micromol/L, median (IQR)	122 (79–199)	84 (59–142)	81 (62–111)	83 (65–117)	85 (64–131)
Minimum platelets, median (IQR)	154 (89–226)	193 (131–270)	193 (144–252)	179 (131–236)	183 (129–248)
Maximum bilirubin in micromol/L, median (IQR)	16 (9–32)	11 (7–20)	10 (7–18)	10 (7–16)	11 (7–19)
Use of any vasopressors, *n* (%)	3,353 (100)	1,760 (22.1)	1,368 (24.6)	3,464 (29.9)	9,945 (34.9)
Sequential Organ Failure Assessment score, median (IQR)	11 (9–14)	6 (4–9)	4 (2–8)	5 (3–9)	6 (3–10)
Outcomes					
IV antibiotics from admission for at least 4 d or until end of ICU stay, *n* (%)	2,778 (82.9)	5,959 (74.8)	2,900 (52.2)	0	11,637 (40.9)
ICU length of stay, d, median (IQR)	6.7 (2.9–14.8)	3.6 (1.8–7.8)	1.8 (0.9–3.5)	2.0 (1.0–4.1)	2.7 (1.1–5.8)
ICU mortality, *n* (%)	954 (28.5)	808 (10.1)	93 (1.7)	676 (5.8)	2,531 (8.9)

ICNARC = Intensive Care National Audit and Research Center, IQR = interquartile range.

Missingness in first 24 hr: maximum heart rate 0.6%, mean arterial pressure less than 0.1%, Fio_2_ 8.2%, Spo_2_ 0.2%, Pao_2_ 6.1%, Pao_2_:Fio_2_ ratio 9.9%, GCS 5.1%, creatinine 4.2%, platelets 4.1%, and bilirubin 16.6%. ICNARC admission diagnosis was missing in 0.7%. All other variables were completely observed.

Over the first 24 hours of admission, the median peak SOFA score was 6 (IQR, 3–10). The most prevalent organ system involvement among ICNARC admission diagnoses (18) was cardiovascular (22.9%), respiratory (21.3%), and gastrointestinal (17.5%). The median length of stay was 2.7 days (IQR, 1.1–5.8 d), and overall ICU mortality was 8.9% (2,531 patients) (Table 1).

### Identification of Sepsis and Septic Shock

**Figure 1.**
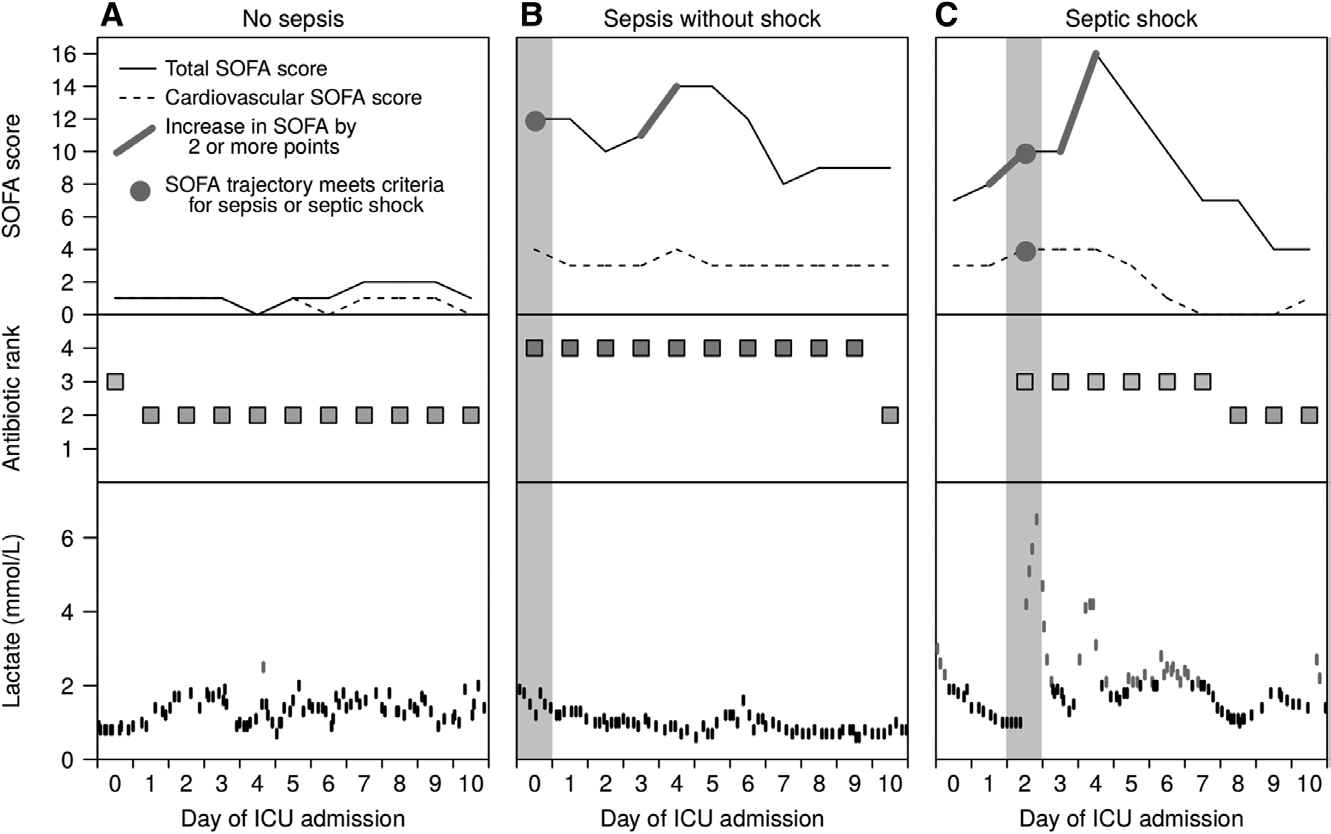
Sample patient timelines showing how physiologic and treatment parameters are tracked over time to enable identification of sepsis (rise in Sequential Organ Failure Assessment [SOFA] score with antibiotic escalation) or septic shock (sepsis with lactate greater than 2 mmol/L and cardiovascular SOFA greater than 2). **A**, Data for a patient who did not have an episode of sepsis. **B**, Data for a patient who was on rank 4 antibiotics and had a high SOFA score at admission, but did not have an elevated lactate, and, therefore, was considered to have sepsis but not septic shock. **C**, Data for a patient who did not have sepsis at admission, but developed a 2-point rise in total SOFA, with cardiovascular SOFA greater than 2, along with a new antibiotic prescription and raised lactate, and hence met the criteria for septic shock on day 2.

**Figure 2.**
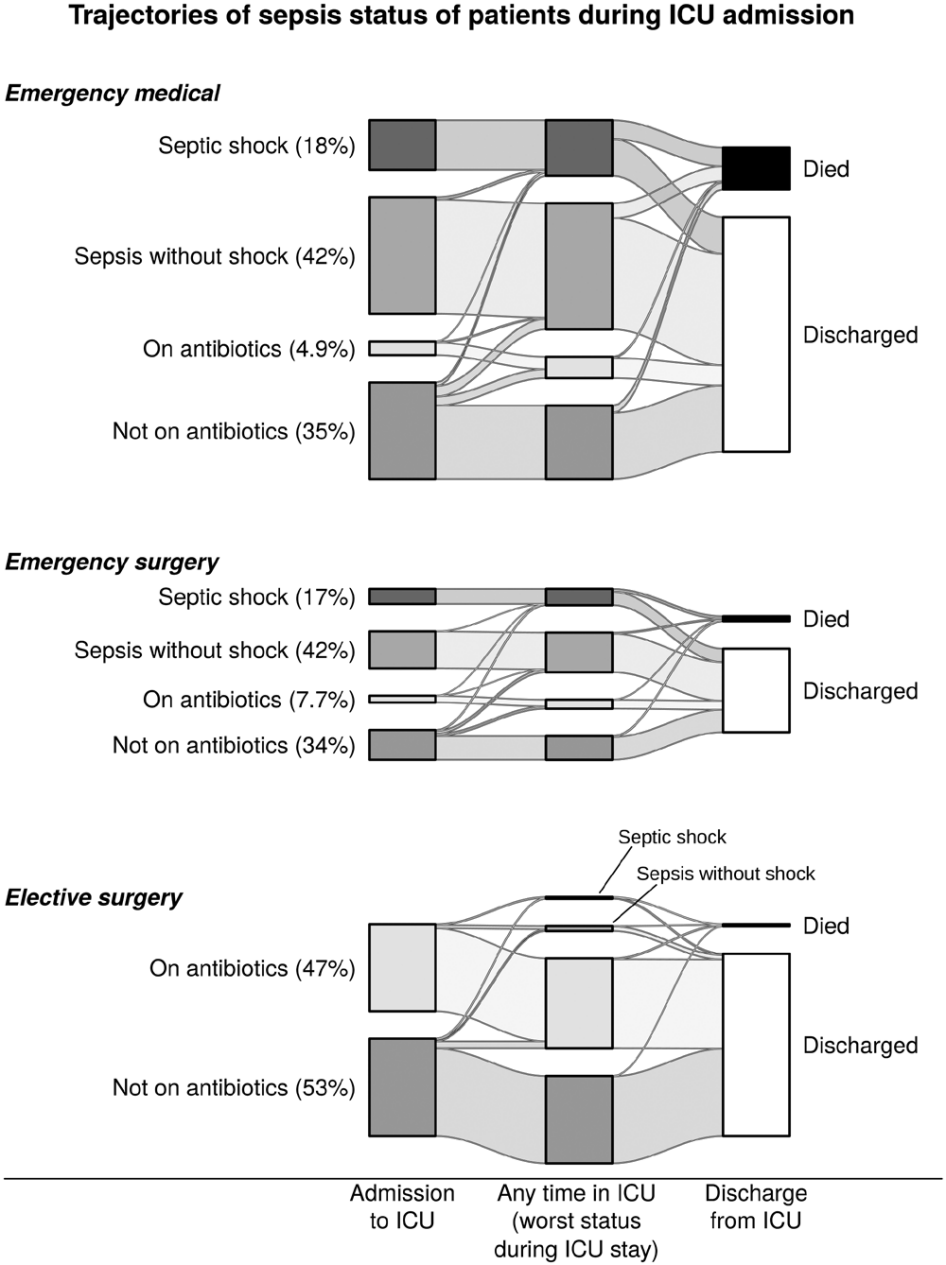
Trajectories of ICU patients by sepsis status at admission.

We identified 29,343 episodes of clinical deterioration where the SOFA score increased by at least 2 points, of which 14,869 (50.7%) were associated with antibiotic escalation and thereby met the Sepsis-3 criteria for sepsis. The majority of sepsis episodes (11,664/14,869, 78.4%) were treated with IV antibiotics for at least 4 days or until the end of the ICU stay. Sample patient trajectories are shown in **Figure 1** The majority of sepsis episodes (76.1%) occurred at admission to ICU; 60.4% of emergency ICU admissions (11,318/18,751) had sepsis, with a similar proportion among emergency medical patients (60.2%) and emergency surgical patients (61.0%) (Table 1 and **Fig. 2**).

Among patients admitted with sepsis, those prescribed IV antibiotics for less than 4 days had 10.2% ICU mortality (264/2,581) and those prescribed antibiotics for at least 4 days or until ICU discharge or death had 17.1% ICU mortality (1,498/8,737) (**sTable 2**, Supplementary Digital Content 4, http://links.lww.com/CCM/G527).

About a quarter of sepsis episodes (4,100, 27.6%) were associated with vasopressor use and lactate greater than 2.0 mmol/L and, therefore, met the criteria for septic shock. The majority of septic shock episodes (85.8%) occurred at admission to ICU; 17.9% of emergency ICU admissions (3,353/18,751) had septic shock, with a similar proportion among emergency medical patients (17.9%) and emergency surgical patients (17.7%). In a sensitivity analysis requiring a minimum of 6 hours for a norepinephrine infusion to be counted for the cardiovascular SOFA, the proportion of emergency ICU admissions with septic shock was 14.6% (2,737/18,751) (**sTable 3**, Supplemental Digital Content 5, http://links.lww.com/CCM/G528).

SOFA scores improved overall after the start of the sepsis episode, in particular the cardiovascular component (**sFig. 2**, Supplemental Digital Content 6, http://links.lww.com/CCM/G529). SOFA scores improved in the days before discharge for those who survived a sepsis episode (**sFig. 3**, Supplemental Digital Content 7, http://links.lww.com/CCM/G530) but deteriorated in patients who died (**sFig. 4**, Supplemental Digital Content 8, http://links.lww.com/CCM/G531). ICU mortality was greater among patients with higher maximum SOFA scores (**sFig. 5**, Supplemental Digital Content 9, http://links.lww.com/CCM/G532). For patients requiring a critical care stay greater than or equal to 1 week, the SOFA score fell from median 9 (IQR, 7–12) on day 1, to 6 (IQR, 4–9) on day 7 (*p* < 0.001 by Wilcoxon rank-sum test).

The overall occurrence rate of sepsis was 83.1 (95% CI, 81.8–84.4) per 1,000 patient days, and for septic shock, 22.9 (95% CI, 22.2–23.6) per 1,000 patient days.

### Community-, Hospital-, and ICU-Acquired Sepsis

The majority of sepsis episodes (12,019, 80.8%) occurred within 48 hours of admission to ICU, of which 7,940 were considered to be community-acquired (in hospital less than 48 hr) and 4,079 hospital-acquired (in hospital greater than 48 hr and in ICU less than 48 hr). The incidence of ICU-acquired sepsis (greater than 48 hr after ICU admission) was 22.4 (95% CI, 21.6–23.2) per 1,000 patient days (2,850 episodes in total), and for ICU-acquired septic shock, 4.48 (95% CI, 4.12–4.85) per 1,000 patient days. Using a stricter definition of sepsis that required at least 4 days of IV antibiotics (unless the patient died or was discharged from ICU), the occurrence rate of ICU-acquired sepsis was 18.7 (95% CI, 18.0–19.5) per 1,000 patient days, and for ICU-acquired septic shock, 4.04 (95% CI, 3.69–4.39) per 1,000 patient days.

Of the 2,040 admissions in which ICU-acquired sepsis occurred, 1,404 (68.8%) had suffered a previous sepsis episode within 48 hours of admission. Compared with hospital- or community-acquired sepsis, ICU-acquired sepsis episodes had a greater relative contribution of CNS SOFA to their delta SOFA score, and a lower contribution from respiratory, renal, coagulation, or liver components (**Table 2**), both in the main analysis and in a sensitivity analysis ignoring GCS measurements on sedation (**sTable 4**, Supplemental Digital Content 10, http://links.lww.com/CCM/G533).

**TABLE 2.** Characteristics of Patients With Sepsis Acquired in the Community, in Hospital, and in the ICU

Source of Sepsis	Community	Hospital	ICU^a^	*p* for Comparison
Number of admissions	7,940	4,079	2,040	
Women, *n* (%)	3,253 (41.0)	1,718 (42.1)	719 (35.2)	< 0.0001
Age, median (IQR)	60.1 (45.4–73.1)	65.1 (51.6–75.5)	61.3 (47.5–72.0)	< 0.0001
Severity of sepsis				
Septic shock, *n* (%)	2,493 (31.4)	1,036 (25.4)	415 (20.3)	< 0.0001
SOFA score, median (IQR)	8 (5–11)	7 (5–11)	9 (6–11)	< 0.0001
Change in component SOFA score on the day that criteria for sepsis were met, mean (sd)				
Cardiovascular	2.28 (1.58)	2.07 (1.58)	1.04 (1.42)	< 0.0001
Respiratory	2.48 (1.11)	2.55 (1.10)	0.87 (1.10)	< 0.0001
Renal	0.84 (1.22)	0.88 (1.21)	0.21 (0.62)	< 0.0001
Coagulation	0.74 (1.01)	0.81 (1.24)	0.14 (0.59)	< 0.0001
CNS	2.05 (1.64)	1.80 (1.56)	1.18 (1.44)	< 0.0001
Liver	0.53 (0.90)	0.55 (0.96)	0.17 (0.54)	< 0.0001
Relative contribution of organ system to overall delta SOFA on the day that sepsis criteria were met (%)				
Cardiovascular	24.7	22.8	26.3	< 0.0001
Respiratory	32.0	34.5	26.6	< 0.0001
Renal	8.8	9.6	6.2	< 0.0001
Coagulation	7.7	8.2	3.6	< 0.0001
CNS	21.6	19.5	32.8	< 0.0001
Liver	5.3	5.4	4.4	< 0.0001
Outcomes				
ICU length of stay, d, median (IQR)	3.8 (1.8–9.0)	4.2 (2.0–9.0)	18.2 (11.1–30.1)	< 0.0001
ICU mortality, *n* (%)	1,022 (12.9)	760 (18.6)	484 (23.7)	< 0.0001
ICU mortality for septic shock, *n* (%)	622 (24.9)	349 (33.7)	170 (41.0)	< 0.0001

IQR = interquartile range, SOFA = Sequential Organ Failure Assessment.

^a^Data are shown for the first episode per admission for ICU-acquired sepsis. Hospital- or community-acquired sepsis could only occur once per ICU admission, by definition. *P* values compare the relevant estimate from community-, hospital-, and ICU-acquired sepsis (proportion tests for categorical variables, Kruskal-Wallis tests for variables with median and IQR quoted, and analysis of variance tests for other variables).

### ICU Mortality

**Figure 3.**
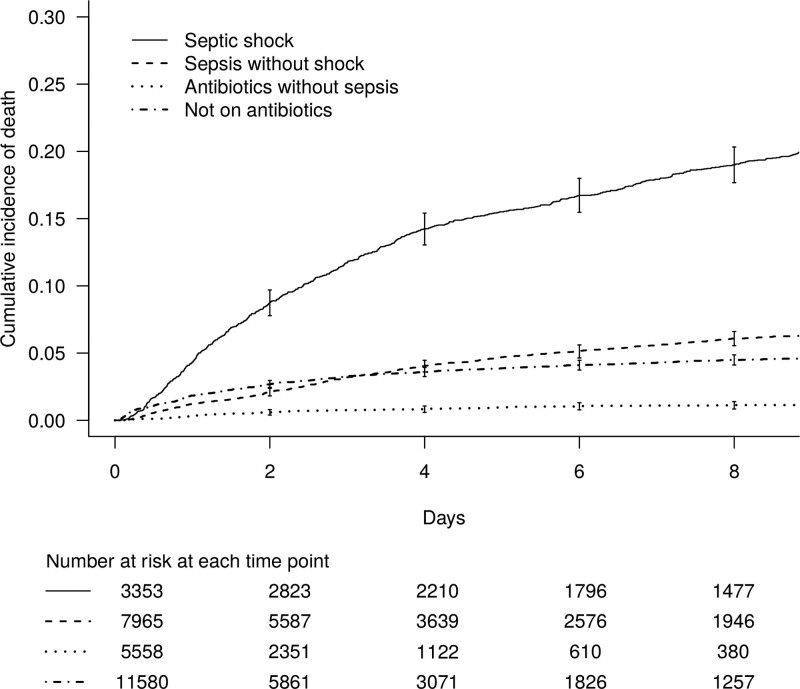
Cumulative incidence curves for all-cause ICU mortality by infection status at admission to ICU.

Seven-day ICU mortality by admission status was highest for those with septic shock (cumulative incidence, 17.9%; 95% CI, 16.6–19.2%), followed by sepsis without shock (5.6%; 95% CI, 5.1–6.1%). It was lowest for patients on antibiotics without sepsis, as the majority of these were elective patients receiving prophylactic antibiotics (**Fig. 3** and Table 1). Among emergency admissions, the subdistribution hazard ratios of death for sepsis without shock (compared with no sepsis), adjusted for age, sex, and admission category, was 0.80 (95% CI, 0.72–0.88), and for septic shock was 1.58 (95% CI, 1.42–1.75) (**sTable 5**, Supplemental Digital Content 11, http://links.lww.com/CCM/G534). Overall ICU mortality for emergency admissions was 2,432/18,917 (12.9%).

ICU mortality by source of sepsis was highest for ICU-acquired sepsis (23.7%; 95% CI, 21.9–25.6%), followed by hospital-acquired sepsis (18.6%; 95% CI, 17.5–19.9%), and community-acquired sepsis (12.9%; 95% CI, 12.1–13.6%) (*p* for comparison less than 0.0001) (Table 2).

### Antibiotic Use

Of the 28,456 critical care admissions studied, 14,188 (49.9%) never received an antibiotic, 9,264 received one antibiotic regimen, and 5,004 received greater than or equal to two antibiotic regimens. The median duration of IV antibiotics per sepsis episode was 4 days (IQR, 2–6 d). Only 19.5% of antibiotic courses (12,283) were for narrow spectrum antibiotics; most were for antibiotics from ranks 2 (broad spectrum, 23,713 courses, 37.6% of total) and 3 (extended spectrum, 20,115 courses, 31.9% of total). The five most common prescriptions were for coamoxiclav (17.4% of antibiotic courses), gentamicin (11.4%), meropenem (7.5%), metronidazole (10.2%), and vancomycin (5.8%) (**sTable 6**, Supplemental Digital Content 12, http://links.lww.com/CCM/G535).

Antibiotic prescribing changed over the course of the patients’ ICU admission; rank 1 and 2 antibiotics (e.g., coamoxiclav, cefuroxime, and metronidazole) tended to be used earlier in the admission, whereas later in the admission, there was more use of rank 3 and 4 antibiotics (e.g., meropenem, amikacin, and vancomycin) (**sFig. 6**, Supplemental Digital Content 13, http://links.lww.com/CCM/G536). We observed a decrease in piperacillin-tazobactam use in early 2017 corresponding with a national shortage (**sFig. 7**, Supplemental Digital Content 14, http://links.lww.com/CCM/G537).

## DISCUSSION

### Summary of Main Findings

In our retrospective analysis of 28,456 ICU admissions between 2014 and 2018 using the NIHR CC-HIC database, using operational criteria for sepsis recommended by the Sepsis-3 Definitions Task Force, we found that sepsis affected around 60% of emergency ICU admissions. Patients admitted with sepsis but no shock had better survival than emergency patients admitted with nonsepsis diagnoses, but patients with septic shock fared much worse. Overall ICU mortality rates for sepsis were consistent with previous studies (3, 22).

The majority of patients with sepsis acquired it prior to ICU admission, but outcomes were worse for patients acquiring sepsis in ICU. This is likely to reflect the increasing pathogenicity of infecting organisms in ICU, increased encounters with multidrug-resistant bacteria, and deteriorating physiologic resilience of the patient.

This is the first U.K. study to use longitudinal EHR data from multiple ICUs to track patients’ physiology and treatments throughout their stay. Use of EHR data to identify sepsis episodes may facilitate future research on sepsis, and if algorithms are implemented real-time within EHRs, they may also facilitate clinical decision support or recruitment to clinical trials (23).

### Classification of Organ Dysfunction

Some of the SOFA definitions use healthcare interventions as a proxy for the physiologic condition of the patient and can be influenced by changes in healthcare practice. Vasopressor administration is used as a proxy for refractory hypotension (i.e., the need for vasopressors), and we classified a higher proportion of sepsis episodes as septic shock if we used the sepsis 3 definitions as published (where the maximum cardiovascular SOFA score in 24 hr would be driven by any use of vasopressors), compared with our sensitivity analysis in which we ignore brief (less than 6 hr) periods of norepinephrine use (sTable 3, Supplementary Digital Content 5, http://links.lww.com/CCM/G528). Similarly, it is unclear how best to use GCS measurements on sedation in calculating the neurologic SOFA (24), but this did not have a major influence on our results (sTable 4, Supplemental Digital Content 10, http://links.lww.com/CCM/G533).

### Comparison With Other Studies

The estimated incidence and outcomes of sepsis depend on the methodology used to identify patients. Recent publications from Australia (25) and the United States (11–14, 22, 26) comparing sepsis incidence and outcome using coding or clinical definitions show marked variation depending on the methodology, which may also be impacted by healthcare policies (27). Definitions applied to contemporaneous EHRs may provide more stable incidence estimates (13).

### Antibiotic Use and Suspected Sepsis

Sepsis is a difficult diagnosis to make contemporaneously, and the sepsis-3 criteria are intended to provide a pragmatic, reproducible definition for clinical and epidemiological purposes. However, the definition relies on the clinical suspicion of infection and, thus, includes patients who were started on antibiotics but subsequently considered not to have sepsis. A Dutch study found only 33% of patients (843/2579) initially treated for sepsis at admission to ICU were subsequently judged to have definite sepsis on manual adjudication (28). Antibiotic duration has been suggested as a way of retrospectively differentiating patients with true sepsis from those in which the initial suspicion of sepsis was not borne out. We found that patients admitted with sepsis given antibiotics for at least 4 days had greater mortality (17.1%) than those in which antibiotics were discontinued within 4 days (10.2%), but our study was limited by lack of antibiotic data on patients discharged from ICU before their antibiotic course had finished.

A broad range of antibiotics was used in this cohort, reflecting the complexity of these cases. We found high reliance on broad and extended spectrum antibiotics (ranks 2 and 3); this likely reflects clinician uncertainty, culture negativity, and desire to cover a broad range of potential pathogens. This becomes more pronounced later on in the patient’s admission, where restricted use antibiotics were used more frequently (sFig. 6, Supplemental Digital Content 13, http://links.lww.com/CCM/G536).

These data also demonstrate the impact of external events. The decrease in piperacillin-tazobactam use in early 2017 (sFig. 7, Supplemental Digital Content 14, http://links.lww.com/CCM/G537) reflects the decrease in drug availability following an earthquake in China (29).

### Limitations of This Study

Our study has a number of limitations. First, we did not have access to the clinical notes that would have enabled direct validation of the sepsis phenotypes by manual review. We also did not have access to the ICD-10 codes entered by clinical coding staff after the admission, but a previous study in one of our units found that only 34% (11/32) of patients with a sepsis ICD-10 code had sepsis according to manual adjudication (10). We defined infection as an escalation in antibiotic therapy and, therefore, did not include sepsis caused by organisms other than bacteria. It may be useful to carry out studies using ICU free-text clinical notes to identify clinical suspicion of infection (23).

Second, the timing and completeness of recording of physiologic parameters were variable. We had to make assumptions about pre-ICU SOFA scores because these data were not available.

Third, we were unable to confirm the presence of infection using microbiology results, but even if these data were available, they would likely underestimate the incidence of infection, as around half of ICU patients with sepsis have no organisms identified on culture (30, 31). We relied on antibiotic prescription as an indicator of infection, but antibiotics are often prescribed empirically in ICU (19, 30), and prescription rates may vary by unit policies and individual clinician behavior (32). The original analysis for developing the Sepsis-3 criteria (3), defined ‘infection, as a new antibiotic prescription with a blood culture, is being taken. Although blood cultures prior to initiation of antibiotics are recommended practice in all our participating units, it is likely that some patients do not receive these investigations (19), and as it is dependent on clinical judgment, it may not add much to the objectivity of ascertaining infection.

Fourth, even if infection and organ dysfunction were confirmed by the algorithm, it is possible that the organ dysfunction was not caused by the infection, and there was an alternative rationale for the antibiotic prescription.

Fifth, we were limited to reporting short-term outcomes within ICU, but future linkage with national registries of deaths and hospital admissions will enable longer term implications of critical illness to be studied. A broader understanding of the epidemiology of sepsis will require the study of patients outside ICU, as the majority of patients with sepsis are not admitted to ICU (9).

Finally, the ICUs in this study were all in academic hospitals that are regional specialist centers, and the patients may not be representative of the general U.K. critical care population. However, our results are consistent with previous studies from other critical care populations (8, 30, 33).

## CONCLUSIONS

We were able to apply the Sepsis-3 criteria for sepsis to an EHR dataset and describe a contemporary population of patients with sepsis in multiple ICUs. Operational definitions based on detailed EHRs may facilitate future studies on sepsis.

## Supplementary Material


