# Supplementary material for: Descriptors of Sepsis Using the Sepsis-3 Criteria: A Cohort Study in Critical Care Units Within the U.K. National Institute for Health Research Critical Care Health Informatics Collaborative*
Source: Crit Care Med. 2021 Jul 1;49(11):1883–94. doi: 10.1097/CCM.0000000000005169 (PMC8508729; doi:10.1097/CCM.0000000000005169)
Supplement: Supplementary file 1 [file ccm-49-1883-s001.pdf]

Supplemental Digital Content 1

Supplementary methods

Data extraction for the CC-HIC database

The data pipeline for the Critical Care Health Informatics Collaborative (CC-HIC) database has been published previously [1], and will be briefly described here. Data were extracted from the electronic health records (EHR) of each ICU (intensive care unit) using bespoke scripts for automated extraction, supplemented by manual extraction if needed. Data were transformed into a custom XML-based format for each ICU to transmit their data to the coordinating centre. This study includes data extracted from Phillips Healthcare and Epic systems, and there is no barrier to extraction from other EHR systems. Future versions of the database will require data to be submitted using HL7 FHIR (Health Level 7 – Fast Healthcare Interoperable Resources, https://www.hl7.org/fhir/), which is a widely adopted international messaging standard for health records.

At the coordinating centre, the XML collection files were checked for quality and completeness, and transformed into a relational database for ease of querying.

Selection of valid critical care episodes for the study

The period of observation for each patient was the ICU admission (‘spell’). The CC-HIC data contains spell-level data items (such as patient demographics, admission and discharge details, and ICNARC diagnoses) and repeated measures for longitudinal data (such as clinical measurements and laboratory data). We used the NHS number to link together ICU admissions that were for the same patient. We excluded patients without a valid NHS number (such as foreign or private patients), as we would not be able to link their admissions together. Some ICU admissions involved patients being moved from one physical ICU to another, and this may be recorded as two separate ICU admissions. We therefore merged together ICU admissions (spells) with fewer than 6 hours between them. This also enabled ICU spells in different CC-HIC hospitals in different Trusts to be linked together, and appear as one admission in our dataset. Each admission was attributed to the site that the patient was admitted to at the start of the admission.

We removed ICU admissions for patients who were aged under 18 at admission, and those with missing sex or cause of death. We excluded incomplete ICU episodes (patients still admitted at the end of the data collection, for whom the outcome was unknown). We excluded ICU admissions where the start and end dates / time overlapped with another admission, as this was likely to indicate an error in the admission dates.

We used data only when the centres were submitting high quality data, as assessed on completeness of SOFA metrics and other parameters. This was assessed by evaluating the completeness of recording of each of the physiological and treatment measures used in SOFA scores over calendar time for each unit. Systematic deficiency in one of these implied that data was not being recorded appropriately, and that period of time was excluded for that unit. The periods of time during which

high quality data was being submitted by each unit is documented in sTable 1 (Supplementary Digital Content 2).

Demographic variables such as age, admission category, discharge status were completely recorded, and admissions with any of these parameters missing were excluded from analysis.

Creation of repeated measures dataset

We created an analysis dataset with each longitudinal variable sampled once per hour. We used these values to calculate the SOFA component scores each hour, as described below. The overall SOFA score, antibiotic usage and sepsis status were calculated for each 24 hour time period from the time of admission (described as an 'day' of ICU admission, which would in most cases straddle two calendar days). We assumed that SOFA scores were zero prior to ICU admission, as per recommendations on the implementation of the sepsis-3 definition which suggests the "baseline SOFA score can be assumed to be zero in patients not known to have pre-existing organ dysfunction" [3].

Missing data

Physiological parameters were recorded according to clinical need and were not necessarily present within every 24 hour period. We excluded patients with data for fewer than 3 SOFA dimensions recorded in the first 24 hours. In the dataset used for analysis, the missingness of recording of physiological parameters in the first 24 hours was as follows: maximum heart rate 0.6%, MAP <0.1%, FiO2 8.2%, SpO2 0.2%, PaO2 6.1%, P:F ratio 9.9%, GCS, 5.1%, creatinine 4.2%, platelets 4.1%, bilirubin 16.6%. ICNARC admission diagnosis was missing in 0.7%.

Implementation of SOFA score

We calculated the SOFA component scores as per the definition of Vincent et al., with a few modifications. We considered each component score to be zero if there were no data recorded to allow calculation of the SOFA component in a 24 hour period. We used the worst SOFA component value in each 24 hour period to calculate the summary score.

Cardiovascular: The cardiovascular component of the SOFA score is defined using mean arterial pressure and the need for vasopressors to maintain adequate blood pressure. Vasopressor use was defined in terms of dosages of dopamine, epinephrine, norepinephrine and dobutamine. Some centres used the drug vasopressin as a vasopressor agent, so we allocated a cardiovascular SOFA score of 4 for patients administered vasopressin at any dose.

Norepinephrine was the most commonly used vasopressor agent, and in some patients it was used for a brief period of time and may not have been strictly necessary. We carried out a sensitivity analysis ignoring administrations of norepinephrine that lasted fewer than 6 hours.

Respiratory: The respiratory SOFA component was calculated using the calculated PaO_2 / FiO_2 ratio and the use of ventilatory support.

Renal: Urine output was not reliably recorded electronically so it was not used for the calculation of this score, instead it was based solely on creatinine measurements.

Coagulation: The coagulation SOFA component was calculated from the platelet count, as per the original description.

Central nervous system: The neurological SOFA component was calculated according to the glasgow coma score (GCS). GCS may be affected by sedative medication, and the original paper describing the SOFA score stated “it is not clear whether the actual or the assumed (in the absence of sedative / relaxant drugs) should be used, so that it was decided to include both, at least initially” [4].

Therefore in the main analysis we used all GCS measurements whether or not the patient was receiving sedative medication. This could have resulted in some false positive physiological deteriorations, when the neurological SOFA score increased because of a change in sedative medication rather than a pathological reason. We carried out a sensitivity analysis in which GCS values on sedative medication were ignored. We assumed that the sedative effect lasted for 24 hours from a record of administration; if there were no GCS measurements off sedation during a 24 hour period it was assumed to be normal.

Liver: The liver SOFA component was calculated from the bilirubin measurement, as per the original description.

Antibiotic use

We used medication administration data to identify use of antibiotics during each 24 hour period. An antibiotic was considered to be ‘on’ during a 24 hour period if the administration occurred within the 24-hour period or within 12 hours before the start of the 24-hour period.

We classified antibiotics according to the ‘ranking’ scale proposed by Braykov et al. [5], which represents their activity against drug-resistant organisms. Rank 1 antibiotics are narrow spectrum (e.g. 1st and 2nd generation cephalosporins, amoxicillin); rank 2 are broad spectrum (e.g. 3rd generation cephalosporins, macrolides, fluoroquinolones and co-amoxiclav); rank 3 are extended spectrum (e.g. anti-pseudomonal penicillins, vancomycin); and rank 4 are restricted use (e.g. anti-pseudomonal carbapenems and colistin) [5].

‘Antibiotic escalation’ was defined as an increase in the maximum rank of out of all current antibiotics from one 24 hour period to the next, or an increase in the number of antibiotics prescribed with the same maximum rank. For the purpose of applying the Sepsis-3 criteria, we defined ‘infection’ as a new course of antibiotics or an escalation in antibiotic therapy, with at least one antibiotic given intravenously.

Implementation of sepsis-3 and septic shock definitions

We defined a new sepsis episode as a 24 hour period during which a new antibiotic administration occurred or the antibiotic rank increased, with at least one of the antibiotics being given intravenously, and the SOFA score increased by at least 2 points between the previous and current, current and subsequent, or previous and subsequent 24 hour periods. We assumed that the SOFA score was zero prior to admission to ICU.

We considered that any antibiotic use on the day of ICU admission for elective surgical patients was prophylactic, and did not classify these patients as having sepsis even if they had a high SOFA score. However, if they subsequently required antibiotic escalation with a rise in SOFA score, they were classified as having sepsis.

We defined a 72 hour period after a sepsis episode during which another sepsis episode could not be identified, as it was likely to be part of the same episode rather than a new infection.

Septic shock was defined as a sepsis episode with cardiovascular SOFA score 3 or greater (i.e. using vasopressors) and a maximum lactate in a 24-hour period of 2 mmol/L. We assumed that vasopressors were administered if required to maintain a mean arterial pressure ≥ 65 mmHg, assuming adequate fluid administration.

Program code

The analysis code is deposited at: https://doi.org/10.5281/zenodo.4089003

The program tree contains the following folders:

- config – YAML files with lookup tables and configuration information. This folder should also contain a 'config/config.R' script which sets up the environment for analysis. This file is not included in the Zenodo archive as it is very specific for the safe haven environment in which the analysis was carried out.
- prep – scripts to extract data from the CC_HIC database and prepare the master dataset for analysis
- analysis – scripts to produce tabular and graphical outputs from the master dataset

The script 'master.R' runs the entire analysis.

The code uses custom R packages 'ccfun' and 'ccdata'.

The ccfun package contains functions for the calculation of organ-specific SOFA scores. It is available from https://github.com/CC-HIC/ccfun.

The ccdata package contains the full CC_HIC data dictionary and is available from https://github.com/CC-HIC/ccdata.

References

1. Harris S, Shi S, Brealey D, et al. Critical Care Health Informatics Collaborative (CCHIC): Data, tools and methods for reproducible research: A multi-centre UK intensive care database. Int J Med Inform. 2018;112: 82–89. doi:10.1016/j.ijmedinf.2018.01.006

2. NIHR Health Informatics Collaborative Metadata Catalogue. [accessed 24 Feb 2021]. Available: https://hic.nihr.ac.uk/metadata

3. Singer M, Deutschman CS, Seymour CW, et al. The Third International Consensus Definitions for Sepsis and Septic Shock (Sepsis-3). JAMA. 2016; 315(8): 801-10. doi: 10.1001/jama.2016.0287.

4. Vincent JL, Moreno R, Takala J, et al; Working Group on Sepsis-Related Problems of the European Society of Intensive Care Medicine. The SOFA (Sepsis-related Organ Failure Assessment) score to describe organ dysfunction/failure. Intensive Care Med. 1996; 22(7): 707-710.

5. Braykov NP, Morgan DJ, Schweizer ML, et al.: Assessment of empirical antibiotic therapy optimisation in six hospitals: an observational cohort study. Lancet Infect Dis 2014; 14: 1220–1227. doi: 10.1016/s1473-3099(14)70952-1

Strobe statement

	Item No	Recommendation	Included in section
**Title and abstract**	1	(*a*) Indicate the study's design with a commonly used term in the title or the abstract	'Cohort study' in title and abstract
		(*b*) Provide in the abstract an informative and balanced summary of what was done and what was found	Abstract
**Introduction**			
Background/ rationale	2	Explain the scientific background and rationale for the investigation being reported	Introduction
Objectives	3	State specific objectives, including any prespecified hypotheses	Introduction, paragraph 3
**Methods**			
Study design	4	Present key elements of study design early in the paper	Methods: Study population
Setting	5	Describe the setting, locations, and relevant dates, including periods of recruitment, exposure, follow-up, and data collection	Methods: Study population
Participants	6	(*a*) Give the eligibility criteria, and the sources and methods of selection of participants. Describe methods of follow-up	Methods: Study population
		(*b*) For matched studies, give matching criteria and number of exposed and unexposed	Not applicable
Variables	7	Clearly define all outcomes, exposures, predictors, potential confounders, and effect modifiers. Give diagnostic criteria, if applicable	Methods: Identification of infection, Methods: Identification of organ dysfunction, Methods: identification of sepsis and septic shock
Data sources/ measurement	8*	For each variable of interest, give sources of data and details of methods of assessment (measurement). Describe comparability of assessment methods if there is more than one group	Methods: Identification of infection, Methods: Identification of organ dysfunction, Methods: identification of sepsis and septic shock
Bias	9	Describe any efforts to address potential sources of bias	Methods: Statistical analysis
Study size	10	Explain how the study size was arrived at	All eligible patients with sufficient data quality were included
Quantitative variables	11	Explain how quantitative variables were handled in the analyses. If applicable, describe which groupings were chosen and why	Methods: Statistical analysis
Statistical methods	12	(*a*) Describe all statistical methods, including those used to control for confounding	Methods: Statistical analysis
		(*b*) Describe any methods used to examine subgroups and interactions	Not applicable
		(*c*) Explain how missing data were addressed	Methods, paragraphs 1 and 2
		(*d*) If applicable, explain how loss to follow-up was addressed	Methods: Statistical analysis
		(*e*) Describe any sensitivity analyses	Methods: Statistical analysis
**Results**			
Participants	13*	(a) Report numbers of individuals at each stage of study—eg numbers potentially eligible, examined	Results: Characteristics of study population

		for eligibility, confirmed eligible, included in the study, completing follow-up, and analysed	
		(b) Give reasons for non-participation at each stage	Results: Characteristics of study population
		(c) Consider use of a flow diagram	sFigure 1
Descriptive data	14*	(a) Give characteristics of study participants (eg demographic, clinical, social) and information on exposures and potential confounders	Results: Characteristics of study population
		(b) Indicate number of participants with missing data for each variable of interest	Results: Characteristics of study population, Table 1; Supplemental Digital Content 1
		(c) Summarise follow-up time (eg, average and total amount)	Results: Characteristics of study population, Table 1
Outcome data	15*	Report numbers of outcome events or summary measures over time	Results: Identification of sepsis and septic shock
Main results	16	(*a*) Give unadjusted estimates and, if applicable, confounder-adjusted estimates and their precision (eg, 95% confidence interval). Make clear which confounders were adjusted for and why they were included	Results
		(*b*) Report category boundaries when continuous variables were categorized	Not applicable
		(*c*) If relevant, consider translating estimates of relative risk into absolute risk for a meaningful time period	Not applicable
Other analyses	17	Report other analyses done—eg analyses of subgroups and interactions, and sensitivity analyses	Supplementary Digital Content: sTables 2 to 5, sFigures 2 to 7
**Discussion**			
Key results	18	Summarise key results with reference to study objectives	Discussion: Summary of main findings
Limitations	19	Discuss limitations of the study, taking into account sources of potential bias or imprecision. Discuss both direction and magnitude of any potential bias	Discussion: Limitations of this study
Interpretation	20	Give a cautious overall interpretation of results considering objectives, limitations, multiplicity of analyses, results from similar studies, and other relevant evidence	Discussion: Comparison with other studies
Generalisability	21	Discuss the generalisability (external validity) of the study results	Discussion: Comparison with other studies and Discussion: Conclusions
**Other information**			
Funding	22	Give the source of funding and the role of the funders for the present study and, if applicable, for the original study on which the present article is based	Acknowledgements
