# Supplementary material for: Descriptors of Sepsis Using the Sepsis-3 Criteria: A Cohort Study in Critical Care Units Within the U.K. National Institute for Health Research Critical Care Health Informatics Collaborative*
Source: Crit Care Med. 2021 Jul 1;49(11):1883–94. doi: 10.1097/CCM.0000000000005169 (PMC8508729; doi:10.1097/CCM.0000000000005169)
Supplement: Supplementary file 2 [file ccm-49-1883-s002.pdf]

Supplemental Digital Content 2

sTable 1

Characteristics of National Health Service intensive care units included in this study

**NHS Trust**	**University College London Hospitals**	**Guy's and St Thomas'**	**Cambridge University Hospitals**	**Imperial College Healthcare**	**Overall**
Start date of contribution	February 2014	February 2014	August 2015	February 2014	February 2014
End date of contribution	April 2018	July 2017	April 2018	January 2017	December 2018
Number of admissions while unit was submitting satisfactory data	8,289	13,326	4,667	2,504	28,786
Number of admissions included in study	8,246	13,139	4,594	2,477	28,456
Number of patients included in study	7,070	11,023	4,152	2,294	24,539
**N (%) of ICU admissions by admission category**					
Elective surgical	3,176 (38.5%)	5,184 (39.5%)	660 (14.4%)	519 (21.0%)	9,539 (33.5%)
Emergency surgical	1,798 (21.8%)	1,410 (10.7%)	872 (19.0%)	505 (20.4%)	4,585 (16.1%)
Emergency medical	3,272 (39.7%)	6,545 (49.8%)	3,062 (66.7%)	1,453 (58.7%)	14,332 (50.4%)
N (%) of ICU admissions with sepsis	2,672 (32.4%)	4,907 (37.3%)	2,466 (53.7%)	1,273 (51.4%)	11,318 (39.8%)
N (%) of ICU admissions coded as infection in ICNARC data	1,498 (18.2%)	2,888 (22.0%)	862 (18.8%)	568 (22.9%)	5,816 (20.4%)
N (%) of sepsis admissions coded as infection in ICNARC data	1,068 (40.0%)	2,316 (47.2%)	723 (29.3%)	436 (34.2%)	4,543 (40.1%)
ICU mortality for admissions with sepsis, N (%)	452 (16.9%)	667 (13.6%)	359 (14.6%)	284 (22.3%)	1,762 (15.6%)

Abbreviations: ICNARC, Intensive Care National Audit and Research Center; ICU, intensive care unit; NHS, National Health Service
