# Supplementary material for: Descriptors of Sepsis Using the Sepsis-3 Criteria: A Cohort Study in Critical Care Units Within the U.K. National Institute for Health Research Critical Care Health Informatics Collaborative*
Source: Crit Care Med. 2021 Jul 1;49(11):1883–94. doi: 10.1097/CCM.0000000000005169 (PMC8508729; doi:10.1097/CCM.0000000000005169)

Supplemental Digital Content 3

sFigure 1

Consort diagram showing selection of patients for the study

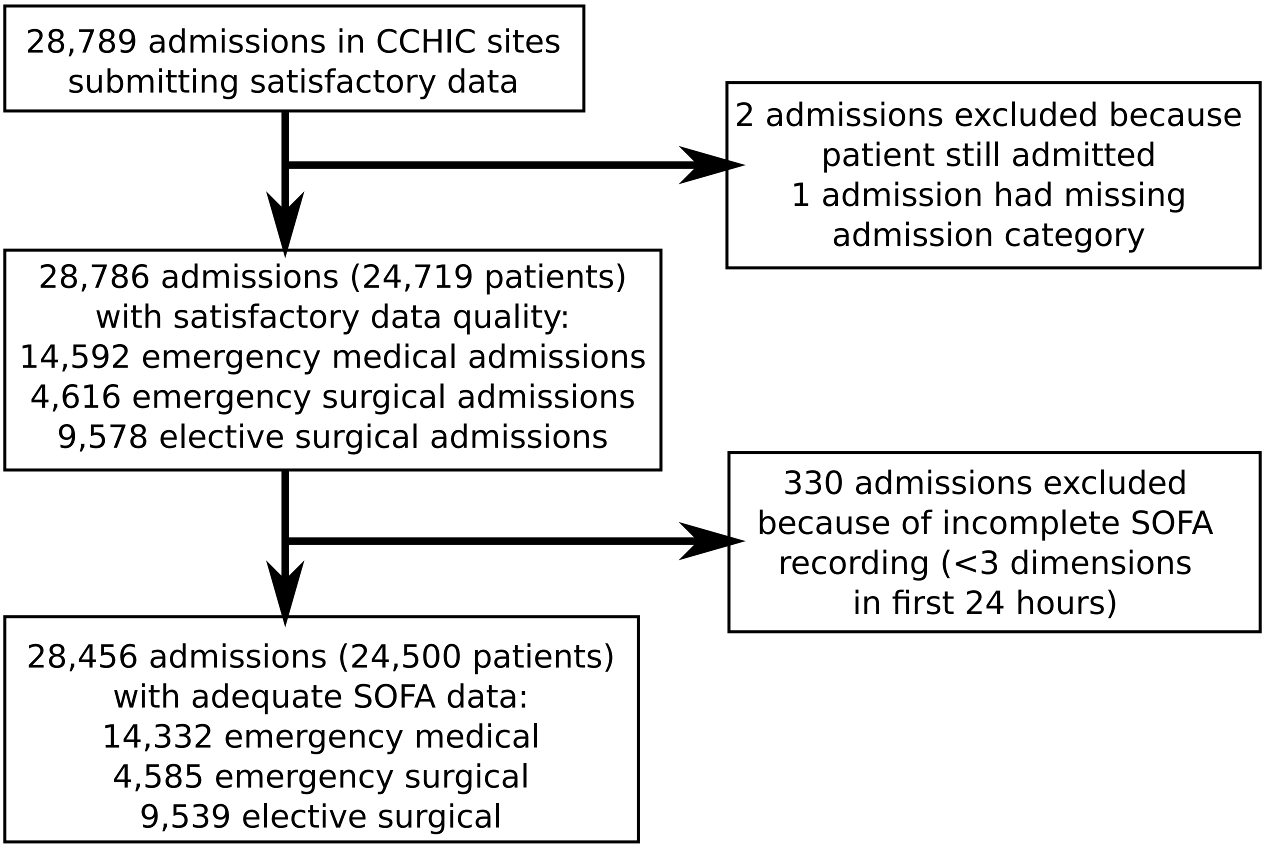

Supplement: Supplementary file 3 [file ccm-49-1883-s003.pdf]
