# Supplementary material for: Descriptors of Sepsis Using the Sepsis-3 Criteria: A Cohort Study in Critical Care Units Within the U.K. National Institute for Health Research Critical Care Health Informatics Collaborative*
Source: Crit Care Med. 2021 Jul 1;49(11):1883–94. doi: 10.1097/CCM.0000000000005169 (PMC8508729; doi:10.1097/CCM.0000000000005169)
Supplement: Supplementary file 4 [file ccm-49-1883-s004.pdf]

Supplemental Digital Content 4

sTable 2

Characteristics of patients admitted to intensive care units with sepsis (as per sepsis-3 criteria) according to duration of intravenous antibiotic administration

Duration of intravenous antibiotics	Antibiotics for at least 4 days or until death	Discharged alive from ICU after < 4 days; total duration of antibiotics unknown	Antibiotics for < 4 days	Overall (all patients with sepsis on admission to ICU)
Number of admissions	5,553	3,184	2,581	11,318
Women, n (%)	2,120 (38.2%)	1,408 (44.2%)	1,176 (45.6%)	4,704 (41.6%)
Age, median (IQR)	62.3 (48.4-73.8)	60.0 (45.0-73.5)	63.8 (48.8-75.5)	61.9 (47.6-74.0)
**Admission category**				
Emergency surgical	859 (15.5%)	1,039 (32.6%)	796 (30.8%)	2,649 (23.8%)
Emergency medical	4,694 (84.5%)	2,145 (67.4%)	1,785 (69.2%)	8,624 (76.2%)
In hospital < 48h prior	2,917 (52.5%)	1,706 (53.6%)	1,573 (60.9%)	6,196 (54.7%)
Septic shock on admission, n (%)	2,318 (41.7%)	575 (22.3%)	460 (14.4%)	3,353 (29.6%)
**Organ system affected on admission (ICNARC admission diagnosis)**				
Cardiovascular	877 (16.0%)	398 (12.6%)	380 (14.8%)	1,655 (14.7%)
Respiratory	2,229 (40.6%)	866 (27.3%)	657 (25.6%)	3,752 (33.4%)
Hematologic	154 (2.8%)	99 (3.1%)	60 (2.3%)	313 (2.8%)
Genito-urinary	405 (7.4%)	426 (13.4%)	238 (9.3%)	1,069 (9.5%)
Neurologic	387 (7.0%)	199 (6.3%)	362 (14.1%)	948 (8.4%)
Gastrointestinal	797 (14.5%)	630 (19.9%)	426 (16.6%)	1,853 (16.5%)
Metabolic or poisoning	150 (2.7%)	202 (6.3%)	148 (5.7%)	500 (4.4%)
Trauma	370 (6.7%)	165 (5.2%)	136 (5.3%)	671 (6.0%)
Other	59 (1.1%)	16 (0.5%)	14 (0.5%)	89 (0.8%)
**First 24h physiology**				
Maximum heart rate, median (IQR)	110 (94-126)	102 (89-116)	100 (87-115)	105 (91-121)
Minimum MAP in mmHg, median (IQR)	61 (55-67)	63 (57-71)	63 (56-70)	62 (56-69)
Maximum FiO2, median (IQR)	0.60 (0.40-0.85)	0.36 (0.28-0.50)	0.40 (0.30-0.60)	0.50 (0.35-0.70)
Minimum SpO2, median (IQR)	91 (88-94)	93 (90-95)	93 (90-95)	92 (89-95)
Minimum PaO2 in mmHg, median (IQR)	7.4 (5.3-9.4)	8.4 (5.6-10.2)	8.5 (5.7-10.1)	7.9 (5.4-9.8)
Minimum P:F ratio, median (IQR)	16 (11-24)	26 (17-38)	24 (15-36)	20 (13-32)
Minimum GCS, median (IQR)	9 (3-14)	14 (10-15)	13 (7-15)	12 (5-15)
Maximum creatinine in micromol/L, median (IQR)	107 (68-193)	81 (59-128)	88 (63-148)	93 (64-166)
Minimum platelets, median (IQR)	174 (102-257)	189 (132-265)	189 (132-252)	182 (1118-258)

Duration of intravenous antibiotics	Antibiotics for at least 4 days or until death	Discharged alive from ICU after < 4 days; total duration of antibiotics unknown	Antibiotics for < 4 days	Overall (all patients with sepsis on admission to ICU)
Maximum bilirubin in micromol/L, median (IQR)	14 (8-27)	11 (7-20)	11 (7-20)	12 (7-23)
Use of any vasopressors, n (%)	3,345 (60.2%)	828 (26.0%)	940 (36.4%)	5,112 (45.2%)
SOFA score, median (IQR)	10 (7-12)	5 (3-8)	7 (4-10)	8 (5-11)
**Outcomes**				
ICU length of stay in days, median (IQR)	8.7 (5.2-16.5)	1.8 (0.9-2.8)	3.1 (2.0-5.7)	4.2 (2.0-9.8)
ICU mortality, n (%)	1,498 (27.0%)	0	264 (10.2%)	1,762 (15.6%)

Abbreviations: GCS, Glasgow Coma Score, ICU, intensive care unit; IQR, interquartile range; ICNARC, Intensive Care National Audit and Research Center; MAP, mean arterial pressure; SOFA, Sequential Organ Failure Score
