# Supplementary material for: Descriptors of Sepsis Using the Sepsis-3 Criteria: A Cohort Study in Critical Care Units Within the U.K. National Institute for Health Research Critical Care Health Informatics Collaborative*
Source: Crit Care Med. 2021 Jul 1;49(11):1883–94. doi: 10.1097/CCM.0000000000005169 (PMC8508729; doi:10.1097/CCM.0000000000005169)
Supplement: Supplementary file 5 [file ccm-49-1883-s005.pdf]

Supplemental Digital Content 5

sTable 3

Sensitivity analysis in which a minimum of 6 hours of norepinephrine administration is required for it to be considered in the calculation of the cardiovascular SOFA: characteristics of ICU admissions by infection status

Infection status on admission	Septic shock	Sepsis without shock	Antibiotics without sepsis	Not on antibiotics	Overall
Number of admissions	2737	8568	5571	11580	28456
Women, n (%)	1,044 (38.1%)	3,656 (42.7%)	2,369 (42.5%)	4,897 (42.3%)	11,966 (42.1%)
Age, median (IQR)	63.5 (49.5-74.3)	61.3 (47-73.9)	63.3 (51.4-72.3)	63.9 (50-74.3)	63.1 (49.3-73.8)
**Admission category**					
Elective surgical	0	0	4,498 (80.7%)	5,041 (43.5%)	9,539 (33.5%)
Emergency surgical	652 (23.8%)	2,038 (23.8%)	356 (6.4%)	1,539 (13.3%)	4,585 (16.1%)
Emergency medical	2,085 (76.2%)	6,530 (76.2%)	717 (12.9%)	5,000 (43.2%)	14,332 (50.4%)
In hospital < 48h prior	1,686 (61.6%)	4,503 (52.6%)	3,071 (55.1%)	6,780 (58.5%)	16,040 (56.4%)
**Organ system affected on admission (ICNARC admission diagnosis)**					
Cardiovascular	646 (24.0%)	1,009 (11.8%)	655 (11.9%)	4,170 (36.2%)	6,480 (22.9%)
Respiratory	748 (27.8%)	3,002 (35.2%)	846 (15.3%)	1,436 (12.5%)	6,032 (21.3%)
Hematologic	63 (2.3%)	250 (2.9%)	30 (0.5%)	140 (1.2%)	483 (1.7%)
Genito-urinary	224 (8.3%)	845 (9.9%)	1,241 (22.5%)	1,332 (11.6%)	3,642 (12.9%)
Neurologic	198 (7.3%)	750 (8.8%)	298 (5.4%)	1088 (9.4%)	2,334 (8.3%)
Gastrointestinal	430 (16.0%)	1,420 (16.7%)	1,729 (31.3%)	1,360 (11.8%)	4,939 (17.5%)
Metabolic or poisoning	59 (2.2%)	441 (5.1%)	156 (2.8%)	1,160 (10.0%)	1,816 (6.4%)
Trauma	263 (9.8%)	401 (4.7%)	64 (1.2%)	556 (4.8%)	1,284 (4.5%)
Other	64 (2.3%)	403 (4.7%)	507 (9.1%)	277 (2.4%)	1,251 (4.4%)
**First 24h physiology**					
Maximum heart rate, median (IQR)	112 (96-128)	103 (90-118)	95 (83-107)	93 (82-105)	97 (85-112)
Minimum MAP in mmHg, median (IQR)	58 (53-63)	63 (57-70)	64 (58-71)	65 (59-73)	63 (57-71)
Maximum FiO2, median (IQR)	0.60 (0.41-0.95)	0.40 (0.30-0.60)	0.35 (0.28-0.45)	0.40 (0.28-0.55)	0.40 (0.28-0.60)
Minimum SpO2, median (IQR)	92 (88-94)	92 (89-95)	94 (92-95)	94 (92-96)	93 (91-95)
Minimum PaO2 in mmHg, median (IQR)	6.3 (4.9-9.2)	8.2 (5.7-10.0)	9.4 (5.7-11.2)	8.8 (5.2-10.8)	8.5 (5.4-10.5)
Minimum P:F ratio, median (IQR)	15 (10-23)	22 (14-33.7)	32 (19.8-44)	26 (15-41)	24 (15-38)
Minimum GCS, median (IQR)	6 (3-13)	14 (7-15)	14 (10-15)	14 (6-15)	14 (6-15)
Maximum creatinine in micromol/L, median (IQR)	126 (81-200)	86 (60-147)	81 (62-111)	83 (65-117)	85 (64-131)
