# Supplementary material for: Descriptors of Sepsis Using the Sepsis-3 Criteria: A Cohort Study in Critical Care Units Within the U.K. National Institute for Health Research Critical Care Health Informatics Collaborative*
Source: Crit Care Med. 2021 Jul 1;49(11):1883–94. doi: 10.1097/CCM.0000000000005169 (PMC8508729; doi:10.1097/CCM.0000000000005169)
Supplement: Supplementary file 6 [file ccm-49-1883-s006.pdf]

Supplemental Digital Content 6

sFigure 2

Distribution of organ-specific Sequential Organ Failure Assessment (SOFA) score components by day after start of sepsis episode

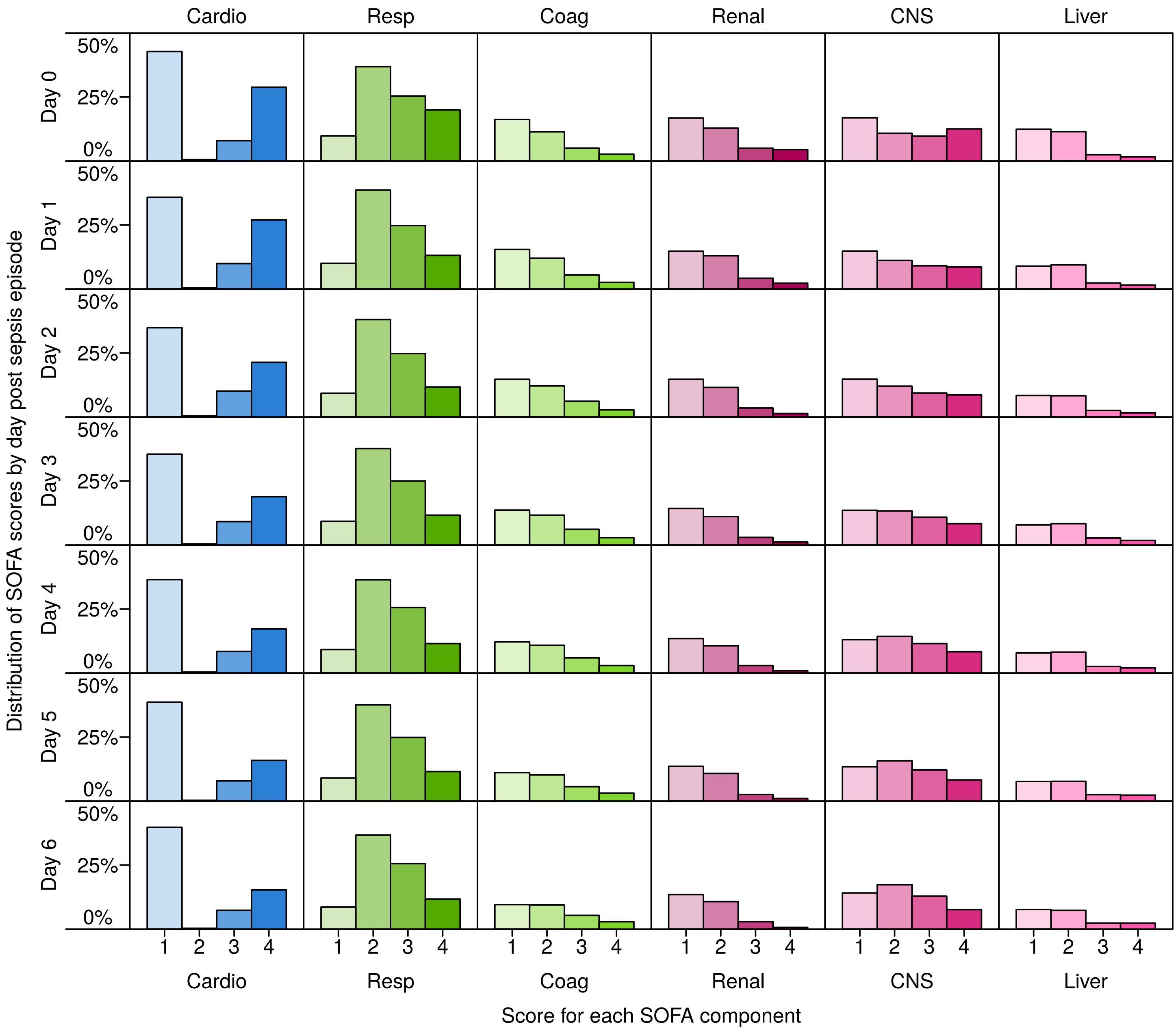

Abbreviations: CNS, central nervous system; Coag, coagulation
