# Supplementary material for: Descriptors of Sepsis Using the Sepsis-3 Criteria: A Cohort Study in Critical Care Units Within the U.K. National Institute for Health Research Critical Care Health Informatics Collaborative*
Source: Crit Care Med. 2021 Jul 1;49(11):1883–94. doi: 10.1097/CCM.0000000000005169 (PMC8508729; doi:10.1097/CCM.0000000000005169)
Supplement: Supplementary file 8 [file ccm-49-1883-s008.pdf]

Supplemental Digital Content 8

sFigure 4

Distribution of Sequential Organ Failure Assessment (SOFA) score components by day prior to death in intensive care unit (ICU), for patients who die in ICU after an episode of sepsis

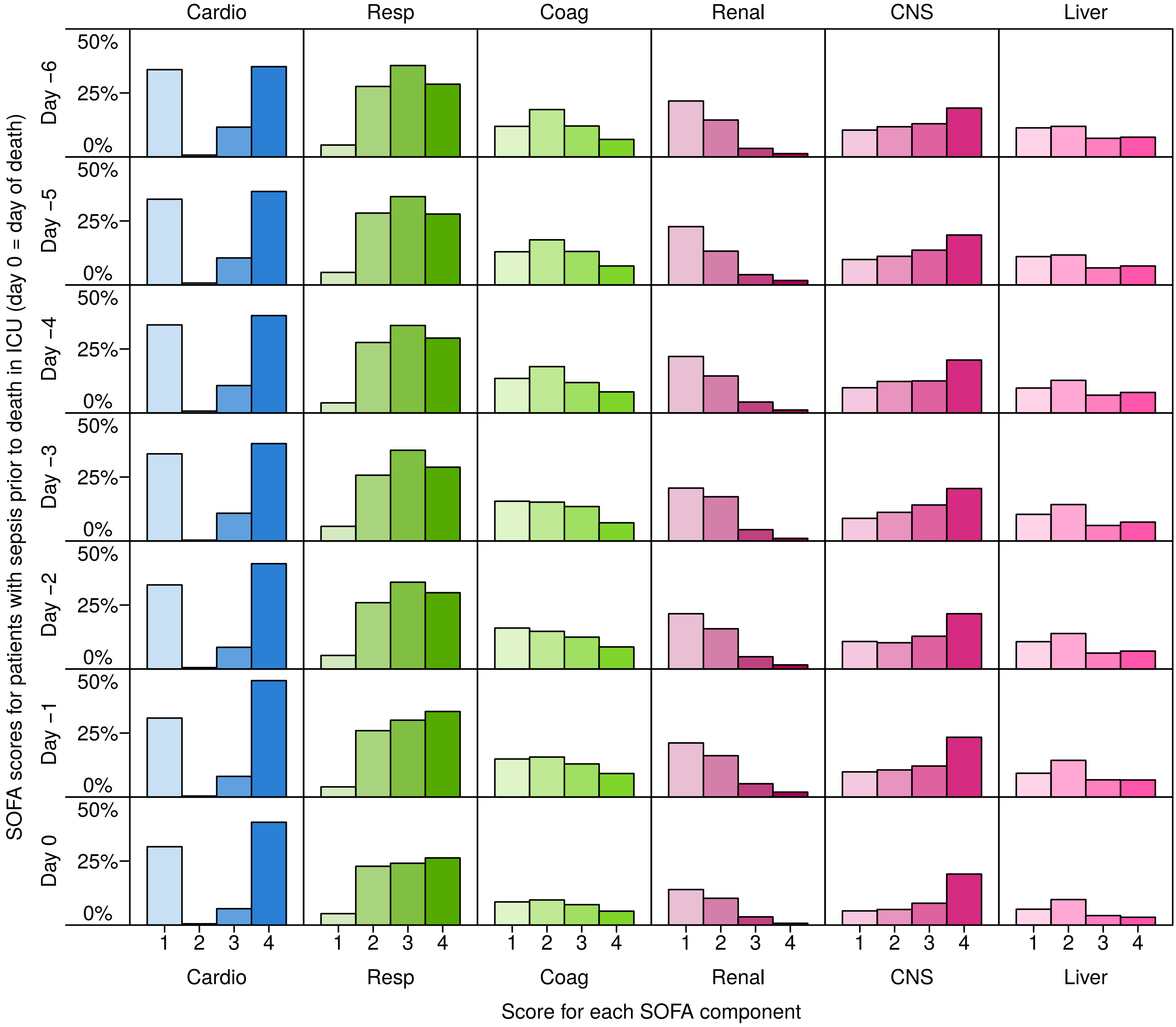

Abbreviations: CNS, central nervous system; Coag, coagulation; ICU, intensive care unit
