# Supplementary material for: Descriptors of Sepsis Using the Sepsis-3 Criteria: A Cohort Study in Critical Care Units Within the U.K. National Institute for Health Research Critical Care Health Informatics Collaborative*
Source: Crit Care Med. 2021 Jul 1;49(11):1883–94. doi: 10.1097/CCM.0000000000005169 (PMC8508729; doi:10.1097/CCM.0000000000005169)

Supplemental Digital Content 9

sFigure 5

All-cause intensive care unit (ICU) mortality (point estimate and 95% confidence interval) by maximum Sequential Organ Failure Assessment (SOFA) score during ICU admission

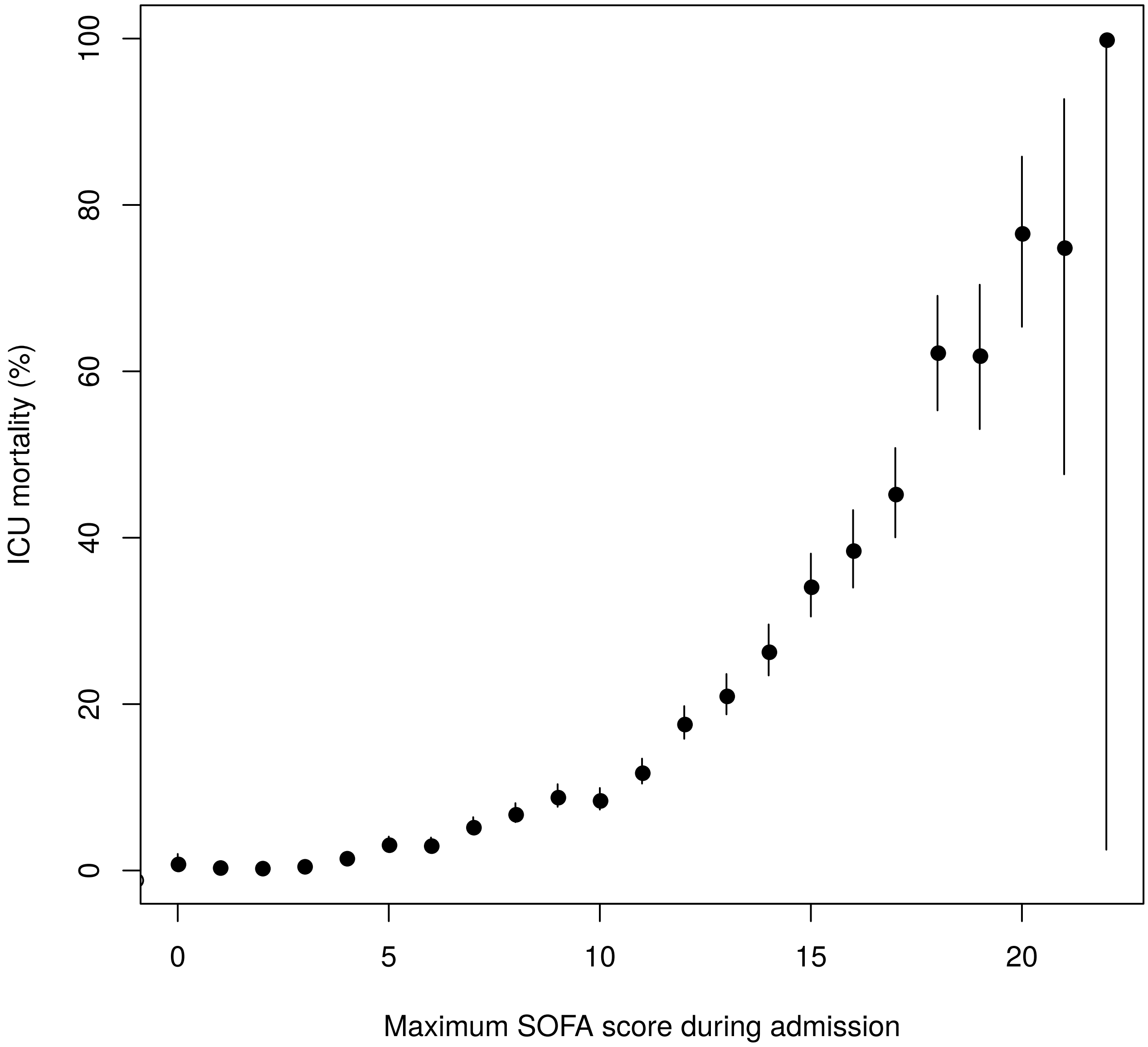

Supplement: Supplementary file 9 [file ccm-49-1883-s009.pdf]
