# Supplementary material for: Descriptors of Sepsis Using the Sepsis-3 Criteria: A Cohort Study in Critical Care Units Within the U.K. National Institute for Health Research Critical Care Health Informatics Collaborative*
Source: Crit Care Med. 2021 Jul 1;49(11):1883–94. doi: 10.1097/CCM.0000000000005169 (PMC8508729; doi:10.1097/CCM.0000000000005169)
Supplement: Supplementary file 10 [file ccm-49-1883-s010.pdf]

Supplemental Digital Content 10

sTable 4

Characteristics and outcomes for patients with sepsis acquired in the intensive care unit, using two methods for handling glasgow coma score values recorded while the patient was receiving sedative medication

Source of sepsis	Using all GCS measurements, regardless of sedation (main analysis)	Omitting GCS measurements on sedation (sensitivity analysis)	*P* value for comparison
N patients	2,040	2,007	
Women, n (%)	719 (35.2%)	717 (35.7%)	0.77
Age, median (IQR)	61.3 (47.5-72.0)	61.5 (48.1-72.1)	0.70
**Severity of sepsis, n (%)**			
Septic shock	415 (20.3%)	403 (20.1%)	0.87
SOFA, median (IQR)	9 (6-11)	7 (5-10)	< 0.0001
**Change in component SOFA score on the day that criteria for sepsis were met**			
Cardiovascular, mean (SD)	1.04 (1.42)	1.07 (1.41)	0.56
Respiratory, mean (SD)	0.87 (1.10)	0.90 (1.09)	0.34
Renal, mean (SD)	0.21 (0.62)	0.22 (0.62)	0.63
Coagulation, mean (SD)	0.14 (0.59)	0.15 (0.60)	0.55
Central nervous system, mean (SD)	1.18 (1.44)	0.85 (1.32)	< 0.0001
Liver, mean (SD)	0.17 (0.54)	0.18 (0.55)	0.47
**Relative contribution of organ system to overall delta SOFA on the day that sepsis criteria were met**			
Cardiovascular, %	26.3	29.2	0.03
Respiratory, %	26.6	28.9	0.06
Renal, %	6.2	6.4	0.72
Coagulation, %	3.6	4.1	0.4
Central nervous system, %	32.8	26.3	< 0.0001
Liver, %	4.4	5.1	0.17
**Outcomes**			
ICU length of stay in days, median (IQR)	18.2 (11.1-30.1)	18.0 (10.9-30.4)	0.78
ICU mortality, n (%)	484 (23.7%)	464 (23.1%)	0.68
ICU mortality for septic shock, n (%)	170 (41.0%)	154 (38.2%)	0.46

Abbreviations: GCS, glasgow coma score; IQR, interquartile range; ICNARC, Intensive Care National Audit and Research Center; SD, standard deviation; SOFA, Sequential Organ Failure Score

Data are shown for the first episode of ICU-acquired sepsis per admission.

P values are from Wilcoxon tests (where median and IQR are quoted), t tests (where mean and SD are quoted) or proportion tests.
