# Supplementary material for: Descriptors of Sepsis Using the Sepsis-3 Criteria: A Cohort Study in Critical Care Units Within the U.K. National Institute for Health Research Critical Care Health Informatics Collaborative*
Source: Crit Care Med. 2021 Jul 1;49(11):1883–94. doi: 10.1097/CCM.0000000000005169 (PMC8508729; doi:10.1097/CCM.0000000000005169)
Supplement: Supplementary file 11 [file ccm-49-1883-s011.pdf]

Supplemental Digital Content 11

sTable 5

Survival models by sepsis status on admission for emergency admissions to intensive care units, showing subdistribution hazards from a Fine and Gray (competing risks) model and cause-specific hazards from a Cox model.

**Sepsis status on admission** (compared to no sepsis)	**Mortality hazard ratio (95% CI)**		**Discharge hazard ratio (95% CI)**	
	**Subdistribution**	**Cause-specific**	**Subdistribution**	**Cause-specific**
Sepsis without shock	0.80 (0.72, 0.88)	0.80 (0.72, 0.88)	0.72 (0.69, 0.74)	0.72 (0.69, 0.74)
Septic shock	1.58 (1.42, 1.75)	1.58 (1.43, 1.74)	0.38 (0.36, 0.40)	0.38 (0.36, 0.40)

Death and discharge alive were modeled as competing outcomes; there was no censoring. Models were adjusted for age, sex, age/sex interaction and admission category (medical or surgical).
