# Supplementary material for: Descriptors of Sepsis Using the Sepsis-3 Criteria: A Cohort Study in Critical Care Units Within the U.K. National Institute for Health Research Critical Care Health Informatics Collaborative*
Source: Crit Care Med. 2021 Jul 1;49(11):1883–94. doi: 10.1097/CCM.0000000000005169 (PMC8508729; doi:10.1097/CCM.0000000000005169)
Supplement: Supplementary file 12 [file ccm-49-1883-s012.pdf]

Supplemental Digital Content 12

sTable 6

Antibiotic use in intensive care units, by rank

Antibiotic	Total number of days	Total number of courses	Percentage of all courses
**Rank 4**			
Meropenem	26,304	4,716	7.5
Amikacin	3,876	1,351	2.1
Linezolid	3,976	716	1.1
Tigecycline	932	110	0.2
Colistin	857	106	0.2
**Rank 3**			
Gentamicin	15,889	7,200	11.4
Piperacillin / tazobactam	28,578	6,317	10.0
Vancomycin	14,278	3,679	5.8
Teicoplanin	6,167	1,969	3.1
Ceftazidime	4,040	915	1.4
Ertapenem	123	35	0.1
**Rank 2**			
Co-amoxiclav	30,603	10,988	17.4
Cefuroxime	7,856	3,759	6.0
Ciprofloxacin	10,882	2,584	4.1
Clarithromycin	7,817	2,550	4.0
Ceftriaxone	3,973	1,241	2.0
Erythromycin	2,744	865	1.4
Clindamycin	2,841	717	1.1
Chloramphenicol	2,120	422	0.7
Levofloxacin	1,068	256	0.4
Azithromycin	826	250	0.4
Moxifloxacin	512	55	0.1
Cefotaxime	55	21	0.0
Ofloxacin	14	5	0.0
**Rank 1**			
Metronidazole	20,847	6,423	10.2
Co-trimoxazole	8,108	1,763	2.8
Flucloxacillin	3,397	1,128	1.8
Doxycycline	2,751	1,031	1.6
Amoxicillin	2,121	602	1.0
Phenoxymethylpenicillin	1,370	311	0.5

Antibiotic	Total number of days	Total number of courses	Percentage of all courses
Benzylpenicillin	1,359	300	0.5
Rifampacin	1,730	222	0.4
Isoniazid	1,317	209	0.3
Pyrazinamide	623	72	0.1
Trimethoprim	171	67	0.1
Ethambutal HCL	412	61	0.1
Nitrofurantion	84	35	0.1
Rifampicin / isoniazid	61	18	0.0
Fusidic acid	57	15	0.0
Rifampicin / isoniazid / pyrazinamide	59	11	0.0
Tobramycin	51	8	0.0
Pentamidine	17	7	0.0
