# Supplementary material for: Descriptors of Sepsis Using the Sepsis-3 Criteria: A Cohort Study in Critical Care Units Within the U.K. National Institute for Health Research Critical Care Health Informatics Collaborative*
Source: Crit Care Med. 2021 Jul 1;49(11):1883–94. doi: 10.1097/CCM.0000000000005169 (PMC8508729; doi:10.1097/CCM.0000000000005169)
Supplement: Supplementary file 13 [file ccm-49-1883-s013.pdf]

Supplemental Digital Content 13
sFigure 6

Relative antibiotic use over the course of intensive care unit admissions

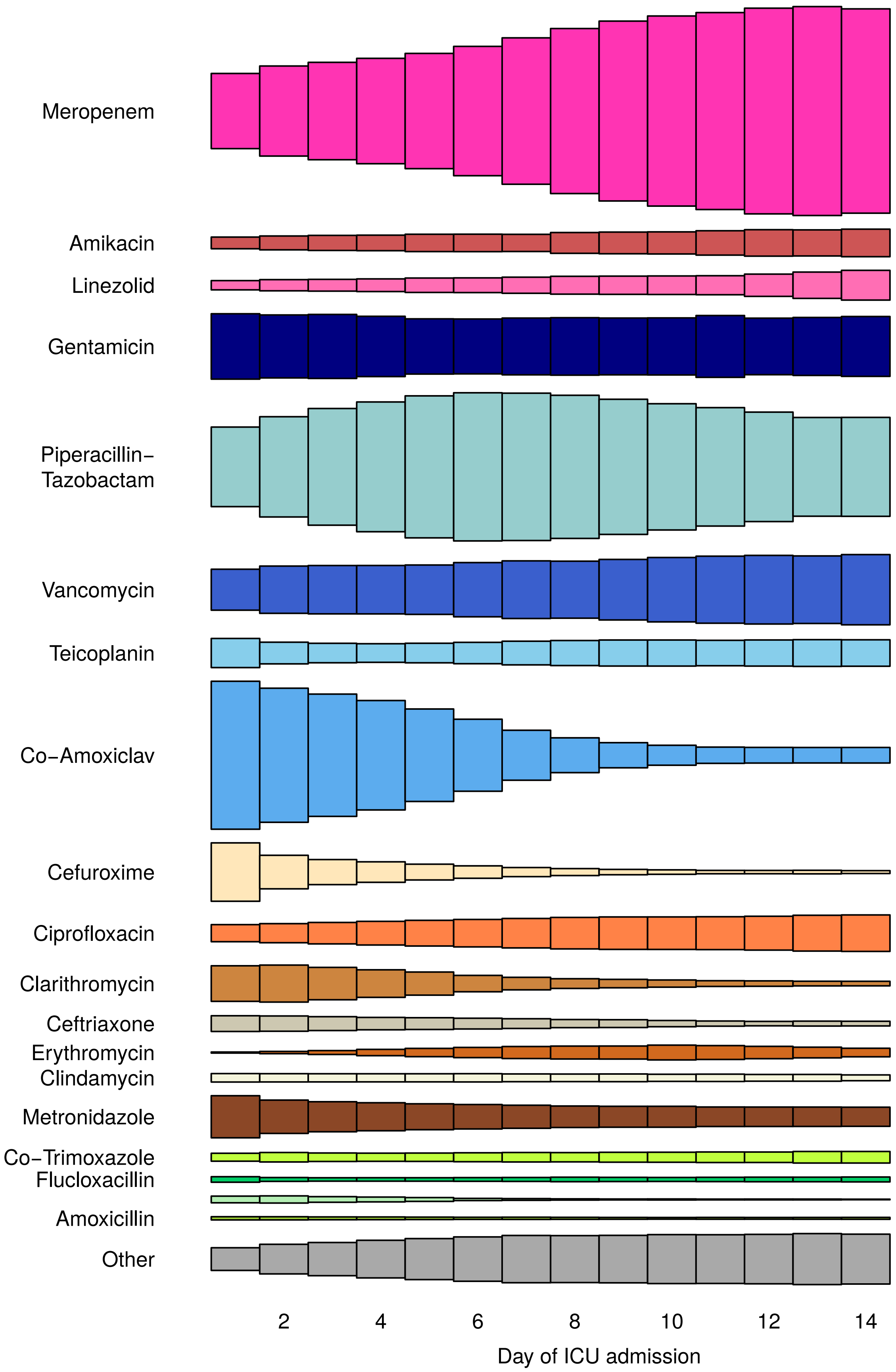

Descriptors of sepsis using the Sepsis-3 criteria: a cohort study in critical care units within the UK NIHR Critical Care Health Informatics Collaborative
